# Report of the M. V. A. D. S.

**Published:** 1852-04

**Authors:** J. Taylor


### REPORT OF THE MISSISSIPPI VALLEY ASSOCIATION OF DENTAL SURGEONS.

In justice to this association, we have reviewed, in part, a report of the society's late proceedings, by Dr. Leslie. The report is entirely too long for insertion in the Register, and particularly so, as the approved minutes of the proceedings have already been published. But as some of the members of the society, as well as many of our subscribers, wish to know something of this "Report," and the review. We shall give quite a number of extracts, embracing principally that part of the "Report" we have to some extent corrected. This we do in justice to the *Reporter*, and that the review may be properly understood.

We first have a few "requests" and "queries" propounded to us by the *Reporter* of the Journal, in relation to plaster impressions, and the "mode upon which the partial vacuum in the chamber is obtained;" and our replies thereto.

The Reporter remarks that

"In answer to the request of Dr. Leslie, that he would explain on scientific principles, the mode upon which the partial vacuum in the chamber is obtained. The Dr. said, he did not know that he could give a full explanation of the thing. About all we know is, that some how or other, it was done by the wearer applying the tongue and sucking out a portion of the air. In answer to another query, the Dr. said, he used the plaster about the thickness of cream. Dr. Leslie further asked, how, in that case, he got the impression of a mouth with a high arch. Dr. Taylor said there was always a portion of plaster in the bottom of the cup a little thicker. This he removed, and with a spatula placed it in the elevated portion of the arch; he then inserted the holder containing the balance."

But in further explanation of plaster impressions, and the method of loosening the same (or wax impresssions also) we have "another thought" which troubles the philosophy of the *Reporter*. He remarks:

"Another thought which has been presented, of which I could not see the philosophy. It has been asserted here, and I have heard of it elsewhere, that if you insert a probe through the plaster or wax, until it touch the elevated portion of the arch, you could immediately remove an impression, which, without such a course, would have been destroyed by the force necessary to remove it at the same period. I say, sir, I cannot understand the working of the probe. It may be ignorance, but it appears to me, that if I was to fill a vessel with wax or plaster, and turn it bottom up, and insert a probe such as the gentleman uses, it would only allow air to pass to the surface the probe touched.

"By what means the air is to pass between the vessel and the wax, and between the gum and wax or plaster, radiating from the small opening, I cannot so clearly see, especially as its effects are said to be immediate. If it were claimed to be a slow process, I would admit it may be of some utility. Or if the plaster or wax had not been placed in close contact with the highest portion of the arch, I could see how the external opening would operate upon the confined air in that cavity, but in the way claimed I cannot. I could see how, if it were such a fluid as water that filled the supposed cup or arch of the mouth, that the insertion of a tube which would pass air to the upper surface would radiate *immediately* over said surface, because in this case, the fluid would immediately descend, not so in the other. In my own practice, consequently, I make no use of the probe. Not because my impressions do not adhere as firmly as those of others, but because I conceive the mode I adopt, of overcoming the difficulty, as superior. My mode of proceedure is based upon what I believe to be a fact, viz. that the soft parts (buccinator muscle and mucous membrane) act the part in the impression that the rim of bukskin does under the air vessel. It makes the adaptation more perfect."

We next have introduced that which aroused most furiously the ire of the *Reporter*. As this does not belong to the approved and adopted minutes of the proceedings of the society, we give this :

" *Resolved,* That this society award to him (Dr. Allen) a gold medal.

" This being seconded by Dr. James Taylor, the president undertook immediately to take the vote. But one of the members desiring to discuss the matter, it was, on his motion, laid on the table until the afternoon session.

" The society then adjourned.

*Afternoon Session.*

" The President announced the first business to be the consideration of the motion laid on the table before adjournment.

" Dr. Taylor desired now to withdraw his second, and offer another resolution as a substitute.

"After some discussion on the points of order involved, further action was, on the suggestion of Dr. Leslie, waived, until the mover, Dr. Goddard, should be present."

We have next the following sly and delicate piece of news. The swallowing of this pill appeared to be necessary to prepare the *Reporter* for that which follows. He remarks that

" Dr. Taylor informed members of the society, that he would, during the coming year, conduct it (the Register) at his own expense, and with this understanding, it was continued under

We shall not pretend to give the *Reporter's* reported speech. He has a right to his own views, so that he does not misrepresent that of others. We pass on therefore to the gentleman's catechism again. This second edition commences with a short speech by myself, and ends with the history of an unfortunate Irishman by "The *Reporter*." He reports me as follows:

"Dr. James Taylor. Mr. President, I am willing to confess that my mind has been changing for sometime on the subject of patents. It is true, I have heretofore stood, as the extracts read by Dr. Leslie show, and I need not refer to them again, to show where I have formeriy stood, Dr. Leslie has done this so well, that I am saved that trouble. My views on the subject of patents, now is, sir, that it would be best to grant this right to the members of the profession, so far as regards the patenting of anything strictly mechanical. But, sir, I would be as much opposed as I ever was to the patenting of any thing not mechancal, to the patenting of anything in our profession, calculated to alleviate the sufferings of poor humanity, in the treatment of disease. The man who would do this, would be unworthy of professional fellowship.

"Dr. Leslie. I would ask the Dr. to explain, when he says he would not agree to the patenting of any thing not mechanical, does he mean that any medicinal compound preparation, or or simple, calculated to remove or relieve disease or suffering in the mouth, should never be patented, but that all of dentistry aside from this may be.

"Dr. Taylor. Yes, sir, that is what I mean. This course may be pursued without injury to our patients, and leaves us room to encourage those who are indefatigable in their labors to improve the practice of dentistry. This principle is allowed by the members of the medical profession. It is but a few days since that I was shown a stethoscope, invented and patented by a practicing physician of Cincinnati.

"It is viewed as a valuable improvement, so much so, that I think no physician will, hereafter, make an examination of the lungs without this improved instrument.

"Dr. Leslie asked, if the inventor is a member in good standing in the medical society.

" Dr. Taylor. I believe he is, and he is known to be the inventor. Besides, this is no new thing for physicians to do. There are trusses innumerable, and very many supporters which have been patented by respectable members of the medical profession, and which are recommended by the eminent physicians.

" There has also been numberless surgical instruments secured to the inventor by patent. So that you see, sir, the position I now stand in; with reference to patenting, is that allowed and upheld by the medical profession.

" That to Dr. Allen should be granted the right to patent, seems to me but justice, having, as he says, devoted a great deal of time and money to the development of this important improvement, for important I believe it to be. It would be expecting too much that a man should devote years of study and labor without a reward, and I think, Mr. President, it is the least we can do in this case, to adopt the proposition of the majority of the committee, and recommend to the *profession* this improvement.

" Dr. Leslie. Mr. President, it makes me smile to hear Dr. Taylor speak as he does, so very ridiculous does his position seem. It reminds me, sir, of the old story of the Irishman's gun. *Now*, the Dr. is willing that if you protect poor suffering humanity from the evil effects that might flow to them from granting to members of our profession the right to patent the medicines they may make use of in dental practice. Why then he rests satisfied. Cut off the right to the patent use of that the Dr. has little use for, very little use for, I may say. As the treatment of disease in the mouth by him or dentists generally, beyond what may be successfully treated by an astringent or stimulant, or the lancet, seldom occurs. Grant this, I say, and he is willing you may patent anything applying to the whole or or a part of the balance.

"And yet, the Dr. would have us believe he is still contending for something grand, something worthy of a liberal member of a liberal profession. It is this makes me smile. It is this reminds me of the Irishman and his gun in need of repair. He said it wanted a new stock, yes, and a lock, aye, and a barrel also; when fully examined, his gun only consisted of the flint. Now, sir, I hold, the Dr. is in a worse plight than the Irishman. Let him deduct from his practice, that portion in which he uses a medicinal preparation and he will find the value of such portion exceedingly small.

"He will, I feel confident, have not enough to form half a flint. Sir, it is idle for him to attempt to lead us astray in this way. He now follows only the *shadow* of a resistance to patents. He has in fact yielded the whole ground he once occupied, having some reason, therefore, which you must have noticed he has not even attempted to state, although I have called upon him for his reasons, and do now urge upon him again, for I have no desire to stand alone in this society. If he has sound reasons and correct motives to influence a change in my action, I pray him to make them known. Until these are produced, I must hold the doctor has made a very lame defence."

In conclusion, we give the Reporter's acknowledgment that he was "desirous of keeping" the gold medal resolution before the society. We refer to page 256 of Journal, and he reports himself as follows:

"But, Mr. President, if Dr. Taylor should suppose, that since the gold medal resolution has been brought before us, I am desirous of keeping it there, let me assure him that, in that he would be correct. I have had no hand, whatever, sir, *in bringing this matter here*, but, since he and his colleagues have put it upon the minutes, I am determined to do all in my power that is proper, to keep it there. I want the profession to know what they have been willing to do; and the Dr. should thank me, that I prevented the vote from being taken so promptly as it was about to be. By my course, he was afforded time for reflection, and saved from a serious mistake. No, gentlemen, seek not to bury the record of your acts. What you have done, if it is right, stand up to it. If it is wrong, acknowledge it, and if you sincerely repent, it may be forgiven. One thing that prompted me to be somewhat tenacious in holding on to correct order, was the improper and unfair means Dr. Taylor made use of to get rid of the resolution. Look at his course in committee. This morning when I reached the hall, no report was shown me until I had asked for it. When obtained, I soon perceived that he had made a change he had no right to do. I found he had copied off the lengthy preamble which had been referred to the committee, certainly an unnecessary labor, the original being plainly written. But his object appeared at the foot of the page. I found that he had, on his own responsibility, expunged Dr. Goddard's resolutions awarding the medal, and to make it look like the original, he had inserted the following: "Therefore, *Resolved*, That we appoint a committee of three, to examine said specimens."

The above extracts are more lengthy than we desired, but our review covered more of "the *Report*" than we at first intended. We know not if we should have noticed it, but the association is misrepresented, and the Register is its proper organ. As the Reporter is desirous "that it may have a wide circulation," *he* at least will not complain.

